# Supplementary material for: Chromothripsis during telomere crisis is independent of NHEJ, and consistent with a replicative origin
Source: Genome Res. 2019 May;29(5):737–49. doi: 10.1101/gr.240705.118 (PMC6499312; doi:10.1101/gr.240705.118)
Supplement: Supplemental Material [file supp_gr.240705.118_Supplemental_file_1.zip › contigs/annotated_contigs/DB115/contig.2.DB115_length_798_mean_cov_13.3458646617.docx]

**DB115_length_798_mean_cov_13.3458646617**

```
AATGCCATGCTAGGGAGTTTAAAGATTTTTGCCAAAATGAAATGATGTCACCAGAAATAGAGCATTACCTGTCTGCCTGGTGTGCATTA
 >chr9:125865028-125865428 + E=9e-229 p=0e+00
GGTGGACAGAAGAGGGAGACATAGCAAGTAGAAGACTGTTTTAATAAATAGACCAGGCAAGAAATTAGATCTTAACTAAAAGCTCCCAA

ATCTTTAGGCATTTCAGGCATTTCTATGCCAGTTGGAGACTGGGGGAATTCCCCGAGGCCTCTCCAGAGTAAATAGACTGCATTCTTGT

CTTGGTTCTCCTCTGGGGAGCCAGTGTCATTGACACCAAGCCCAGCATCCTCATGCACACTGTTCCTCTTGCAGGACTTGGAACACCAT

TTAGGGCTTGCCCTCAATGAGGTGCAGGCAGCCAAGAAGACGTG|AA|AAACTGGGTAATTAGGTTGCGGCCATGCCAGGGAGTTTGGA
                                               >chr9:125868704-125869100 + E=2e-226
GCTGATAAGGAGTCACTGGAGAGTTTTAACCCATTTATGCCAGAGGTTACAAATTTTTTTGTGTGAAAAATCAGACCTTGGCGATGACC

TTAAGCAGTAGGATATAAATAACTCCCACAAGCTTAGCGTTCCAATAATGGAACACTAGGCATAAATGGGTTAAGCTAACTTGCACTTT

GAGTGGTTCCCTGCTGATCCTTTAGGAAGAAAAATCGGGAGGAGAAGAAATTAAGGGTGTGAGCAGAGGTGACAGATGATAGAATAAGG

AATGGAGAACGGAGTCAGTGAAATTTAGGGAGAGTGACGACACACCAGGAAGATGATAAATTTAGCTGTCGGGGTATAGATTTGAAAT
```
